# Substance P and patterned silk biomaterial stimulate periodontal ligament stem cells to form corneal stroma in a bioengineered three-dimensional model

**DOI:** 10.1186/s13287-017-0715-y

**Published:** 2017-11-13

**Authors:** Jialin Chen, Wei Zhang, Peyman Kelk, Ludvig J. Backman, Patrik Danielson

**Affiliations:** 1 Department of Integrative Medical Biology, Anatomy, Umeå University, SE-901 87 Umeå, Sweden; 2 Department of Community Medicine and Rehabilitation, Physiotherapy, Umeå University, Umeå, Sweden; 3 Department of Clinical Sciences, Ophthalmology, Umeå University, Umeå, Sweden

**Keywords:** PDLSCs, Corneal stroma, Substance P, Aligned silk membrane, Differentiation

## Abstract

**Background:**

We aimed to generate a bioengineered multi-lamellar human corneal stroma tissue in vitro by differentiating periodontal ligament stem cells (PDLSCs) towards keratocytes on an aligned silk membrane.

**Methods:**

Human PDLSCs were isolated and identified. The neuropeptide substance P (SP) was added in keratocyte differentiation medium (KDM) to evaluate its effect on keratocyte differentiation of PDLSCs. PDLSCs were then seeded on patterned silk membrane and cultured with KDM and SP. Cell alignment was evaluated and the expression of extracellular matrix (ECM) components of corneal stroma was detected. Finally, multi-lamellar tissue was constructed in vitro by PDLSCs seeded on patterned silk membranes, which were stacked orthogonally and stimulated by KDM supplemented with SP for 18 days. Sections were prepared and subsequently stained with hematoxylin and eosin or antibodies for immunofluorescence observation of human corneal stroma-related proteins.

**Results:**

SP promoted the expression of corneal stroma-related collagens (collagen types I, III, V, and VI) during the differentiation induced by KDM. Patterned silk membrane guided cell alignment of PDLSCs, and important ECM components of the corneal stroma were shown to be deposited by the cells. The constructed multi-lamellar tissue was found to support cells growing between every two layers and expressing the main type of collagens (collagen types I and V) and proteoglycans (lumican and keratocan) of normal human corneal stroma.

**Conclusions:**

Multi-lamellar human corneal stroma-like tissue can be constructed successfully in vitro by PDLSCs seeded on orthogonally aligned, multi-layered silk membranes with SP supplementation, which shows potential for future corneal tissue engineering.

**Electronic supplementary material:**

The online version of this article (doi:10.1186/s13287-017-0715-y) contains supplementary material, which is available to authorized users.

## Background

The cornea is the outermost transparent tissue of the eye, which is important for light refraction and protection of the eye from outside insults and infections. The stroma is the main part of the cornea and is important in its function [1]. The corneal stroma consists of orthogonally aligned arrays of heterotypic collagen type I (COL I) and V (COL V) fibrils, as well as keratocytes, the major cell type in normal cornea. Human corneal stromal stem cells (CSSCs) have been identified in the limbal part of the stroma [2, 3] and are thought to have a potential advantage in future clinical applications for corneal repair by bioengineering corneal tissues from these stem cells [3]. However, the limitation in donors of corneal tissue, the sensitive location of CSSCs, and the complicated components of culture medium [2, 4] are challenges that need to be faced in such bioengineering tasks. Therefore, it is of worth to find alternative stem cells from non-corneal tissues.

A number of stem cells from non-corneal tissues have been reported for corneal repair or keratocyte differentiation, including bone marrow-derived mesenchymal stem cells (BM-MSCs) [5], adipose-derived stem cells (ASCs) [6, 7], and umbilical cord-derived mesenchymal stem cells (UMSCs) [8]. Dental stem cells such as dental pulp stem cells (DPSCs) and periodontal ligament stem cells (PDLSCs) are attracting more and more interest for ocular lineage differentiation and ocular regenerative medicine [9]. The similarity in neural crest origin and proteoglycan secretion between PDLSCs and keratocytes make PDLSCs a potential alternative for corneal repair or keratocyte differentiation [9]. Nevertheless, there have been few studies on this reported so far.

Orthogonally aligned collagen fibrils are one of the most important features of the stroma. Our previous study has combined patterned silk membrane and dome-shaped mechanical stimulation to construct a biomimetic three-dimensional corneal model which is useful for keratocyte culturing in vitro since the model preserves the keratocyte phenotype and alignment [10]. Many other studies have reported the promotion effect of aligned scaffolds on the differentiation of stem cells towards keratocytes, as well as the construction of corneal stroma-like tissue in vitro, with cells of keratocyte phenotype and aligned collagen deposition [3, 11–14]. However, the single layer of stroma-like tissue is still quite far away from the orthogonal multi-layer normal stromal tissue seen in vivo [3, 13]. Although multi-lamellar stroma-like tissues have also been successfully constructed by using various stem cells, the long culture period (from 4 to 9 weeks) [11, 12, 14] hampers their future application.

As one of the most innervated tissues in the body, the physiology and pathology of the cornea is highly regulated by various neurotrophic factors [15–17]. Substance P (SP) is an important neuropeptide. The role of SP has been reported on keratocyte migration [18], corneal epithelial cell apoptosis [19], and epithelial wound healing [20]. The effect of SP on osteoblastic differentiation has also been well studied [21, 22]. However, it is not known if SP has any effects on the differentiation of any kind of cells towards a keratocyte phenotype.

The aim of our current study was to generate a bioengineered multi-lamellar corneal stroma-like tissue in vitro by differentiating PDLSCs towards keratocytes on an aligned silk membrane. The effect of SP on the keratocyte differentiation and stroma tissue construction was evaluated as well. We report that multi-lamellar corneal stroma-like tissue can be constructed successfully in vitro by PDLSCs seeded on orthogonally aligned, multi-layered silk membranes with SP supplementation, which shows potential for future corneal tissue engineering.

## Methods

### PDLSC isolation

In this study, PDLSCs from three individuals were obtained from surgically removed impacted third molars collected from patients at the University Hospital of Umeå. Written informed consent was obtained from all patients. Collection, culture, storage, and usage of all clinical isolates were approved by the Regional Ethical Review Board in Umeå (2013-276-31 M), and were in accordance with the principles of the Declaration of Helsinki.

The periodontal ligament belongs to a specialized connective tissue that connects and anchors the tooth to the alveolar bone and basically outlines the root surface of the tooth. The periodontal tissue was scraped from the root surface by scalpel, cut into pieces (approximately 3–4 mm^3^), and digested with 3 mg/ml collagenase type I (Worthington Biochemicals Corp.) and 4 mg/ml dispase II (Roche Diagnostics) at 37 °C for 1 h. Cells were passed through a 70-μm strainer (BD Falcon™ Labware) to obtain single-cell suspensions, and subsequently cultured in growing medium consisting of minimum essential medium-α (MEM-α with GlutaMax; Life technologies, 32561) with 10% fetal bovine serum (FBS; Life technologies, #16000) and 1% penicillin-streptomycin (Life technologies, #15410). Cells between passages 3 and 6 were used in this study.

### Clone formation assay

Cultured PDLSCs were detached by 0.05% trypsin-EDTA (Life technologies, #25300). To form colonies, 300 cells were seeded per well in a six-well plate. After 10 days of culture, colonies were formed and stained with 1% crystal violet (Sigma, C3886) for 10 min. Colonies with diameters > 2 mm was counted and the ratio was calculated.

### Flow cytometry

According to the manufacturer’s protocol (BD Bioscience), PDLSCs were incubated with PE-conjugated antibodies, directed to CD73, CD90, and CD105, and negative markers cocktail (includes CD11b, CD19, CD34, CD45, and HLA-DR). PE-conjugated isotype-matched IgGs (BD Bioscience) were used as controls. The samples were analyzed using FACS Caliber (BD Bioscience), with 5000 cells chosen for each analysis.

### Multi-lineage differentiation

The differentiation potential of PDLSCs towards the osteogenic and adipogenic lineage was evaluated according to previous reports [23, 24]. PDLSCs were cultured in osteogenic differentiation medium for 4 weeks, and then stained with Alizarin red. Positive induction of adipogenesis was confirmed by Oil Red O staining after 4 weeks of adipogenic induction.

The differentiation potential of PDLSCs towards the teno-lineage was evaluated in an in vitro cell sheet differentiation model as previously described [24]. After confluence, PDLSCs were cultured in Dulbecco’s modified Eagle’s medium (DMEM; Life technologies, 11960) with 10% FBS and 50 μg/ml l-ascorbic acid 2-phosphate (A2-P; Sigma-Aldrich, A8960). Medium was changed every 2 days. After 3 and 7 days of culture, mRNA was extracted for quantitative polymerase chain reaction (qPCR) evaluation.

### Keratocyte differentiation with inducing medium

Keratocyte differentiation of PDLSCs was induced with keratocyte differentiation medium (KDM) as described by Syed-Picard and collaborators [11]. KDM was prepared in advanced DMEM (Life technologies, 12491) supplemented with 1 mM A2-P, 10 ng/mL of basic fibroblast growth factor-2 (bFGF-2; Invitrogen, PHG6015) and 0.1 ng/mL of transforming growth factor-beta3 (TGF-β3; Sigma-Aldrich, SRP3171). After seeding, PDLSCs were cultured in growth medium for 2 days before switching to KDM. The differentiation medium was changed every 2 days. To evaluate the differentiation efficacy of KDM on PDLSCs, cultured primary human limbal keratocytes (at passage 2) were used as controls in the qPCR assay. Primary human limbal keratocytes were isolated and cultured, and have been characterized by our group [25]. Briefly, corneal epithelial and endothelial cells were removed by scraping. The remaining limbal corneal stroma was cut into pieces and digested with collagenase overnight. After centrifugation, the cells were cultured in DMEM/F-12 (Gibco, #21331-046) supplemented with 2% FBS and 1% penicillin-streptomycin. Cells at passage 2 were collected for RNA extraction and followed by qPCR assay.

To evaluate the effect of substance P on keratocyte differentiation, PDLSCs were cultured in KDM with or without 1 μM SP (Sigma, S6883). The medium was changed every second day.

### RNA extraction, cDNA reverse transcription, and qPCR

RNA extraction and cDNA reverse transcription was performed as previously described [16]. qPCR was carried out using SYBR® Green reagents (Applied Biosystems, 4385612) or TaqMan Gene Expression Assay (Applied Biosystems). All primers and probes used in this study are summarized in Tables 1 and 2. Representative results of cells from at least two individuals are displayed as target gene expression normalized to the housekeeping gene.

**Table 1:** Primers used for quantitative polymerase chain reaction

Genes	5′–3′	Primers
Glyceraldehyde 3-phosphate dehydrogenase (*GAPDH*)	Forward	TGACGCTGGGGCTGGCATTG
	Reverse	GGCTGGTGGTCCAGGGGTCT
Scleraxis (*SCX*)	Forward	CGAGAACACCCAGCCCAAAC
	Reverse	CTCCGAATCGCAGTCTTTCTGTC
Mohawk (*MKX*)	Forward	GAAGGCAACTTTGTCTATCGCA
	Reverse	TGATCTCCTTCCAATACGTGTC
Collagen type I (*COL I*)	Forward	CGATGGATTCCAGTTCGAGTAT
	Reverse	CATCGACAGTGACGCTGTAGG
Collagen type XIV (*COL XIV*)	Forward	AAGGATTGCCCTCCGACTACAC
	Reverse	CTGATGCGTTCATTGCCTTCTC
Nuclear factor of activated T-cells 4 (*NFATC4*)	Forward	AAGGGTGAGACGGACATCG
	Reverse	CCGCCCATTGGAGACATAA
Biglycan (*BGN*)	Forward	GATGGCCTGAAGCTCAA
	Reverse	GGTTTGTTGAAGAGGCTG
EPH receptor A4 (*EPHA4*)	Forward	AGTGGGCTGTGACAATCTGGAATA
	Reverse	CATTTAGACGGAACTGAGGAGGGT

**Table 2:** Probes used for quantitative polymerase chain reaction

Gene name	Gene symbol	Assay ID
Lumican	*LUM*	Hs00929860_m1
Keratocan	*KERA*	Hs00559941_m1
Aldehyde dehydrogenase 3A1	*ALDH3A1*	Hs00964880_m1
Aldehyde dehydrogenase 1A1	*ALDH1A1*	Hs00946916_m1
Collagen type I	*COL I*	Hs00164004_m1
Tachykinin precursor 1	*TAC1*	Hs00243225_m1
Tachykinin receptor 1	*TACR1*	Hs00185530_m1
Collagen type III	*COL III*	Hs00943809_m1
Collagen type V	*COL V*	Hs00609133_m1
Collagen type VI	*COL VI*	Hs00915125_m1
MMP1	*MMP1*	Hs00899658_m1
MMP3	*MMP3*	Hs00968305_m1
MMP12	*MMP12*	Hs00899662_m1
MMP14	*MMP14*	Hs01037009_g1
β-Actin	*ACTB*	4352667

### Immunofluorescence

Cultured cells or frozen sections of multi-lamellar constructs were fixed in 4% (v/v) paraformaldehyde. The samples were permeabilized with 1% Triton X-100 and blocked with 1:20 diluted normal serum. Primary antibody (Table 3) was used to incubate the samples at 4 °C overnight. After washing, fluorescein-conjugated secondary antibody (Table 3) was added for 45 min at 37 °C, and DAPI was used to reveal the nuclei of the cells. The integrated density of fluorescence in the different groups was quantified using ImageJ analysis software (NIH).

**Table 3:** Antibodies used for immunofluorescence staining and Western blot

Antibody	Company	Code
Collagen type I	Abcam	ab34710
Collagen type III	Abcam	ab7778
Collagen type V	Santa Cruz	sc-166155
Collagen type VI	Abcam	ab6588
Lumican	Santa Cruz	sc-166871
Lumican	Abcam	ab168348
Keratocan	Santa Cruz	sc-66941
Keratocan	Bioss	bs-11054R
β-Actin	Cell Signal	4967
Polyclonal swine anti-rabbit immunoglobulins/TRITC	Dako	R0156
Polyclonal rabbit anti-mouse immunoglobulins/TRITC	Dako	R0270
Anti-mouse IgG, HRP-linked antibody	Cell Signal	7076
Anti-rabbit IgG, HRP-linked antibody	Cell Signal	7074

### Western blot analysis

Samples were harvested at desired time points. Cells from three replicate wells were collected together as one sample. The protein was extracted and the concentration was quantified. Western blot analysis was performed as previously described [16]. Antibodies used for Western blot analysis are presented in Table 3.

### Scaffold fabrication

Flat and patterned silk membranes (14 mm in diameter) were fabricated as previously described [10]. Groove density at 600 mm^–1^ was used for the preparation of patterned silk membrane. Structures of both flat and patterned silk membranes were observed under scanning electron microscope (SEM) as previously reported [10].

### Cell growth on scaffolds

F-actin staining was performed by incubation for 30 min with BODIPY FL Phallacidin (Invitrogen, B607) to reveal the cell alignment on different silk membranes.

To evaluate the growth and direction of cells on membranes, 1.5 × 10^4^ cells per membrane were seeded and observed under a light microscope every day. Pictures at days 1, 4, 7, and 14 after keratocyte differentiation were taken.

### Orientation angle analysis

To analyze cell alignment, Image-Pro Plus 6.0 software (Media Cybernetics) was used. For each analyzed cell, the orientation angle was calculated by measuring the difference in the angle between the longest axis of the cell and the silk grooves. In each sample, 20 representative cells were measured for statistical analysis.

### Aspect ratio analysis

Image-Pro Plus 6.0 software (Media Cybernetics) was used to determine the aspect ratio of the cells. For each analyzed cell, the length and width was measured. The ratio of length to width was calculated to show the cellular aspect ratio. In each sample, 20 representative cells were measured for statistical analysis.

### Construction of multi-lamellar corneal stroma tissue in vitro

To construct multi-lamellar corneal stroma tissue in vitro, 2 × 10^5^ cells were seeded on each patterned silk membrane and cultured in growing medium for 24 h. Six cell-seeded membranes were orthogonally stacked one by one in a 24-well plate, with two plastic rings on the top of the construct to keep the membranes close together. The constructs were cultured in KDM with 1 μM SP for 18 days, with the medium changed every 2 days; samples were then collected. Frozen sections (12 μm) were prepared using a microtome and subsequently stained with hematoxylin and eosin (H&E) or antibodies for immunofluorescence observation.

### Statistical analysis

Statistical data are shown as mean ± SD unless specially declared. Student’s *t* test was performed for two-group comparison. One-way analysis of variance (ANOVA) with Bonferroni post-hoc test was performed for comparison of more than two groups. All experiments were performed in triplicate and were successfully repeated in PDLSCs derived from different patients. For all comparisons, *p* < 0.05 was considered statistically significant.

## Results

### Isolation and identification of PDLSCs

**Fig. 1:**
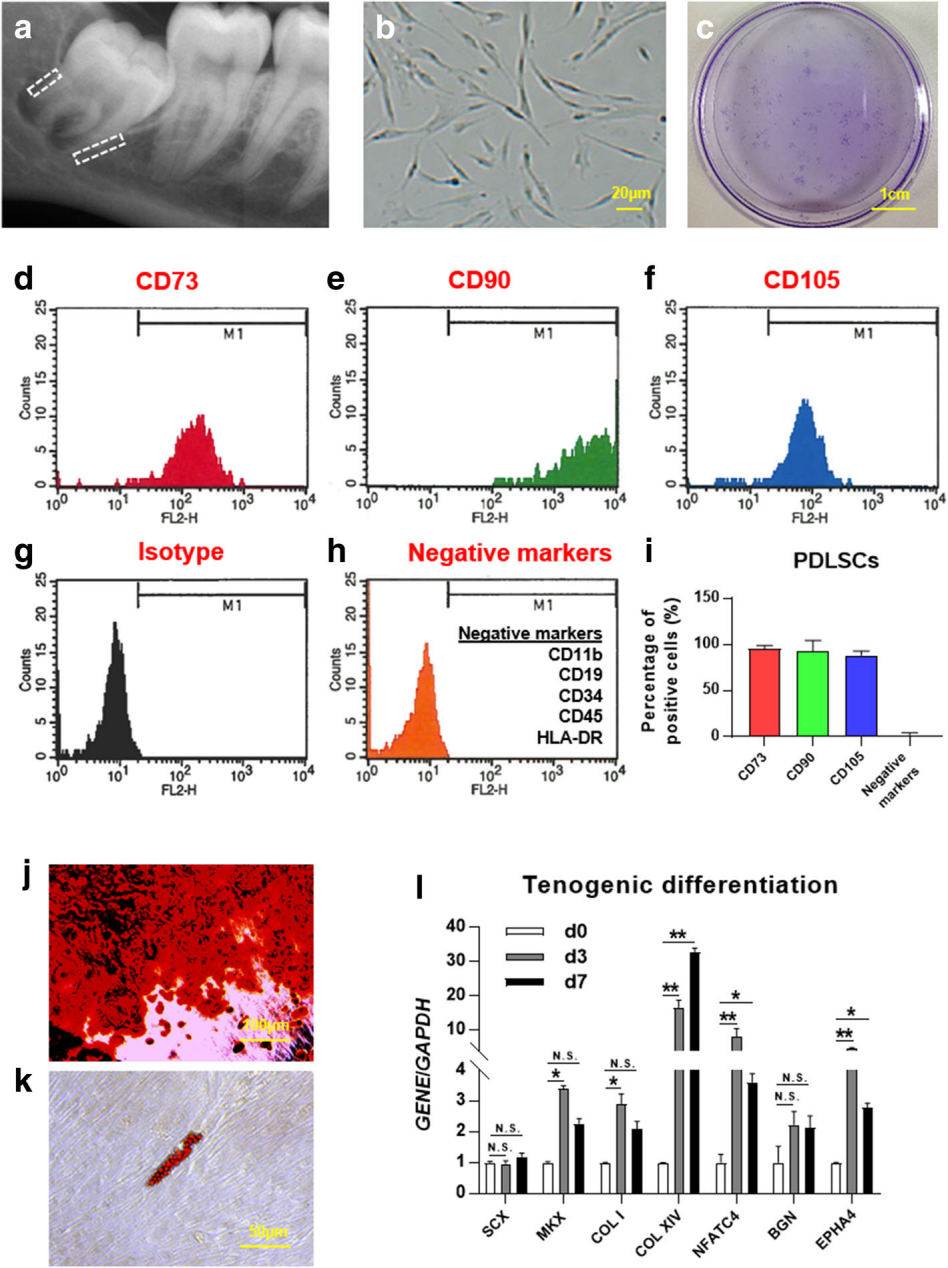
Isolation and identification of periodontal ligament stem cells (*PDLSCs*). **a** Representative intraoral radiograph. The sites of isolation for human PDLSCs are highlighted by the *dashed boxes*. **b** Cell morphology under a light microscope. **c** Clones formed from a single-cell culture after 10 days. **d**–**i** Expression profile of MSC surface markers in PDLSCs, as compared to isotype control. Data are from PDLSCs derived from three different individuals. **j** Alizarin red staining after 4 weeks of osteogenic induction. **k** Oil Red O staining after 4 weeks of adipogenic induction. **l** Tenogenic differentiation in a cell-sheet model. Gene expression of tendon-related genes was evaluated by qPCR. Levels at day (d)0 were set as 1 in the quantified data, and all other groups were compared to d0 in the statistical analysis. *p < 0.05, **p < 0.001. *N.S.* not significant

Human PDLSCs were isolated from surgically removed impacted third molars without completed root formation (Fig. 1a). After expansion, the cells displayed fibroblast-like morphology (Fig. 1b). The clonogenic ability of PDLSCs was evaluated by low-density seeding of cells on the culture plate. After 10 days of culture, 9.89 ± 1.26% cells were found to form clones by crystal violet staining (Fig. 1c). These PDLSCs expressed the same surface CD markers as mesenchymal stem cells (MSCs), including CD73 (Fig. 1d), CD90 (Fig. 1e), and CD105 (Fig. 1f), as compared to isotype control (Fig. 1g). They were negative for CD11b, CD19, CD34, CD45, and HLA-DR (Fig. 1h). A similar high expression profile to MSC CD markers (CD73, CD90, and CD105) were found in PDLSCs isolated from three different individuals (Fig. 1i) and these were used in the following experiments. The differentiation potential of PDLSCs was also determined. Osteogenic differentiation assays showed the existence of mineralized calcium deposits after 4 weeks of induction, as confirmed by Alizarin red staining (Fig. 1j). The adipogenic differentiation potential of these cells was shown by the accumulation of lipid droplets (around 1% of total cells) after 4 weeks of induction (Fig. 1k). The tenogenic differentiation potential of PDLSCs was evaluated in a cell-sheet model. A significant increase was found in the expression of tendon-related genes, including *MKX*, *COL I*, *COL XIV*, *NFATC4*, and *EPHA4* (Fig. 1l).

### PDLSCs differentiate into keratocytes with induction medium

**Fig. 2:**
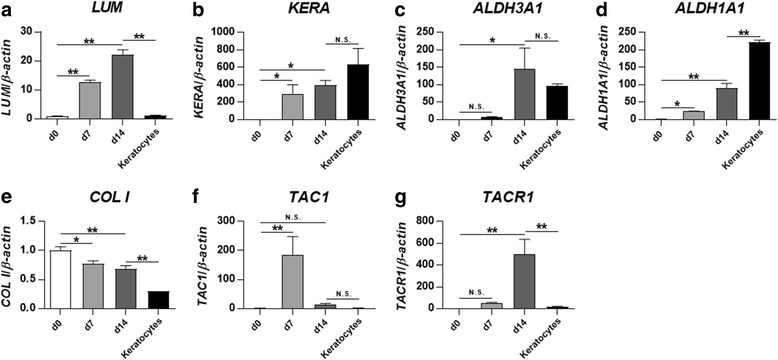
PDLSCs differentiate into keratocytes with induction medium. Keratocyte differentiation medium was used to differentiate PDLSCs towards keratocytes for 7 and 14 days. The mRNA levels of *LUM* (**a**), *KERA* (**b**), *ALDH3A1* (**c**), *ALDH1A1* (**d**), *COL I* (**e**), *TAC1* (**f**), and *TACR1* (**g**) were evaluated by qPCR. Representative results are shown from PDLSCs derived from two different individuals. Levels at day (d)0 were set as 1. The expression was compared between d0 and d7, d0 and d14, and d14 and primary in-vitro cultured normal keratocytes. *p < 0.05, **p < 0.001. *N.S.* not significant

Keratocyte differentiation medium (KDM) was used to differentiate PDLSCs towards keratocytes. After 7 and 14 days of induction, the gene expression of the keratocytes markers lumican (*LUM*), keratocan (*KERA*), aldehyde dehydrogenase 3A1 (*ALDH3A1*), and aldehyde dehydrogenase 1A1 (*ALDH1A1*) was compared to day 0 and to primary in-vitro cultured normal keratocytes (Fig. 2a–d). Up to a 100-fold increase in expression was found during the differentiation. It was noticed that after 14 days of induction the expression levels were comparable with (*KERA*, *ALDH3A1*, and *ALDHA1*) or even higher (*LUM*) than that of primary in-vitro cultured normal keratocytes. The expression of collagen type I (*COL I*), the main extracellular matrix (ECM) component of corneal stroma, was decreased slightly after induction (Fig. 2e). To explore the potential role of SP on the keratocyte differentiation of PDLSCs, the expression of *TAC1* (the gene coding for SP) was analyzed, as was the gene for the SP preferred receptor, *TACR1* (coding for the neurokinin-1 receptor). Interestingly, both of these genes showed major change during the differentiation process (Fig. 2f and g).

### Substance P promotes collagen expression during induced keratocyte differentiation

**Fig. 3:**
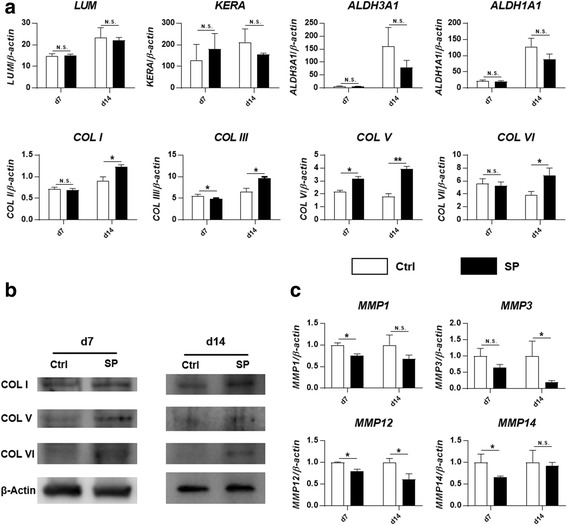
Substance P (*SP*) promotes collagen expression during induced keratocyte differentiation. Keratocyte differentiation medium was used to differentiate PDLSCs towards keratocytes for 7 and 14 days, with (SP) or without (*Ctrl*) substance P. **a** Gene expression was evaluated by qPCR. Representative results are shown from PDLSCs derived from three different individuals. Levels at day (d)0 were set as 1. **b** Western blot was carried out to compare the collagen expression between control and SP-treated group. No bands of COL III with predicted size were found in our blots. **c** Gene expression of *MMPs* was evaluated by qPCR. Levels of the control group were set as 1. *p < 0.05, **p < 0.001. *N.S.* not significant

To detect the effect of SP on keratocyte differentiation of PDLSCs, the expression of keratocyte markers was compared between PDLSCs treated with KDM supplemented with SP and PDLSCs treated with KDM alone. No significant difference was found for any of the genes *LUM*, *KERA*, *ALDH3A1*, or *ALDH1A1* at either day 7 or day 14 after induction (Fig. 3a). However, SP promoted gene expression of collagens, especially after 14 days of induction (the main types of collagen in the stroma: *COL I*, 1.35-fold, *p* < 0.05; *COL V*, 2.16-fold, *p* < 0.001; minor types of collagen in stroma: *COL III*, 1.46-fold, *p* < 0.05; *COL VI*, 1.81-fold, *p* < 0.05) (Fig. 3a). This effect was further confirmed by Western blot (Fig. 3b) and immunostaining (Additional file 1: Figure S1) at the protein level, with more collagen deposition in the SP-treated group as compared to the control group. The role of SP in promoting collagen expression could be attributed to its effect of decreasing *MMP* expression. SP treatment significantly reduced the expression of *MMP1*, *MMP3*, *MMP12*, and *MMP14*, either at day 7 or at day 14 after induction (Fig. 3c). *MMP2* and *MMP9* were detected as well, but no differences were found (data not shown).

### Patterned silk membrane guides cell alignment

**Fig. 4:**
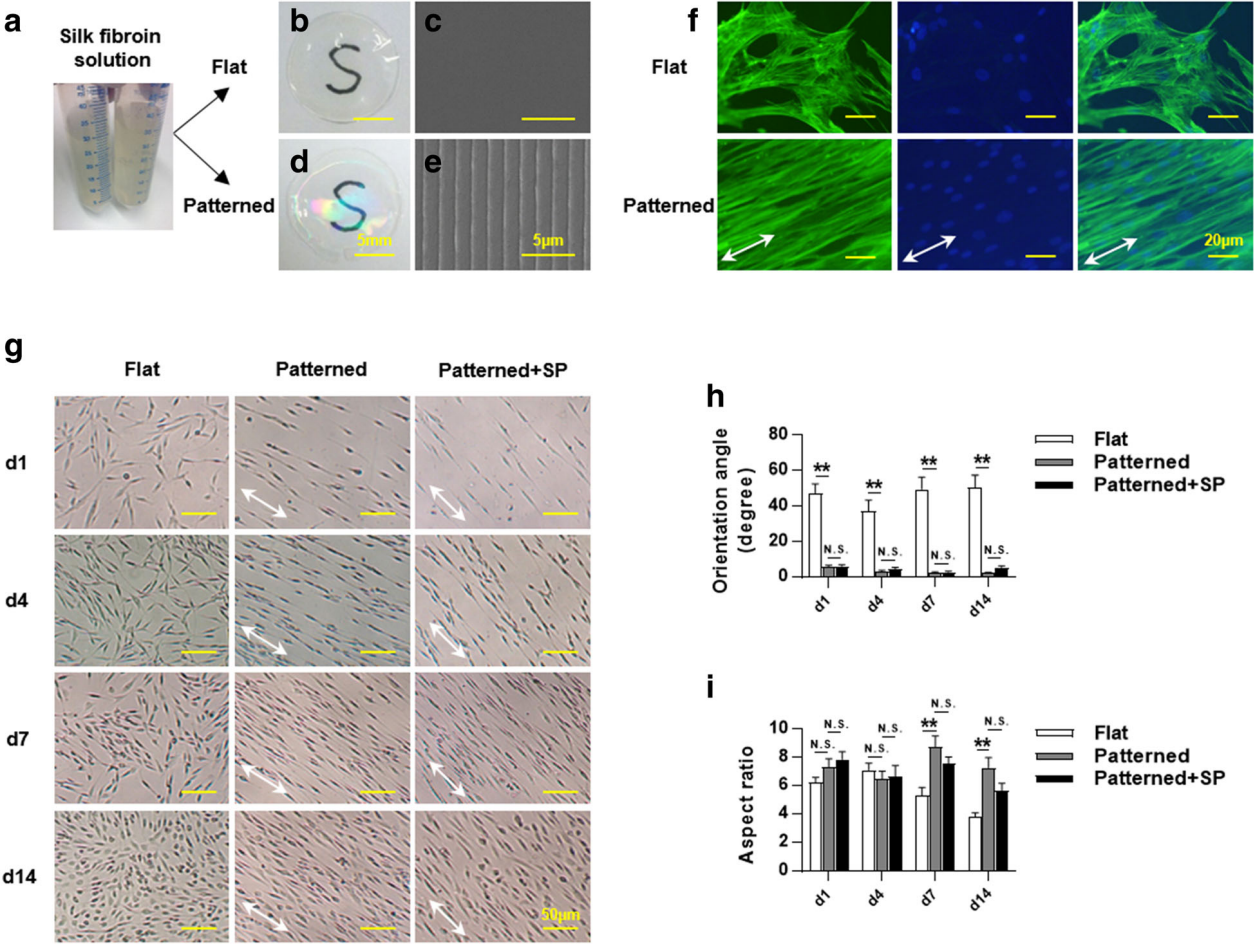
Patterned silk membrane guides the cell arrangement. Silk fibroin solution (**a**) was used to fabricate flat (**b** and **c**) and patterned (**d** and **e**) silk membranes. **b, d** Macroscopic pictures show that the silk membranes were clear enough to allow visualization of the letters below. **c, e** SEM pictures revealed the surface morphology of the membranes. **f** The cell alignment on silk membranes were compared by F-actin staining. The *right panels* show the merged picture of the *left panels* (F-actin staining) and the *middle panels* (DAPI staining). *White arrows* indicate the direction of the grooves on patterned silk membranes. **g** PDLSCs were seeded and differentiated by induction medium on silk membranes with or without substance P (*SP*). Pictures were taken at day (d) 1, 4, 7, and 14 after induction. *White arrows* indicate the direction of the grooves on patterned silk membranes. Cellular orientation angles (**h**) and cellular aspect ratios (**i**) of seeded cells were determined. All other groups were compared to patterned silk membranes at each time point in the statistical analysis. Results are shown as mean ± SEM. **p < 0.001. *N.S.* not significant

Flat and patterned (aligned) silk membranes were fabricated using silk fibroin solution as previously reported [10] (Fig. 4a). Both of these were smooth and transparent (Fig. 4b and d). The surface morphology of the membranes was revealed under SEM (Fig. 4c and e). F-actin staining showed more cell alignment on patterned silk membrane as compared to flat silk membrane (Fig. 4f). PDLSCs were seeded and differentiated by induction medium on flat silk membrane, patterned silk membrane, or patterned silk membrane supplemented with SP. The growing and arrangement of cells were continuously observed (Fig. 4g). The results showed that all three groups support cell growth and amplification on the silk membranes. Cells were randomly arranged on flat silk membranes, but were directed along the axis of the patterned silk membranes. No obvious difference was found between the control or SP-treated groups under light microscopy. Quantification of the cellular orientation angle further confirmed that patterned silk membranes significantly enhanced cell alignment at each time point, as compared to flat silk membranes (Fig. 4h; *p* < 0.001). There was no significant difference with or without SP supplementation (*p* ≥ 0.05). The aspect ratio analysis showed that there was no significant difference in cell extremities between patterned silk membranes and flat silk membranes at day 1 and 4 (Fig. 4i; *p* ≥ 0.05). However, the cells cultured on patterned silk membranes were more elongated as compared to those on flat silk membranes at day 7 and 14 (*p* < 0.001). No significant difference was found between patterned silk membranes alone or the SP-supplemented group at each time point (*p* ≥ 0.05).

### Stromal ECM deposits on patterned silk membranes with substance P

**Fig. 5:**
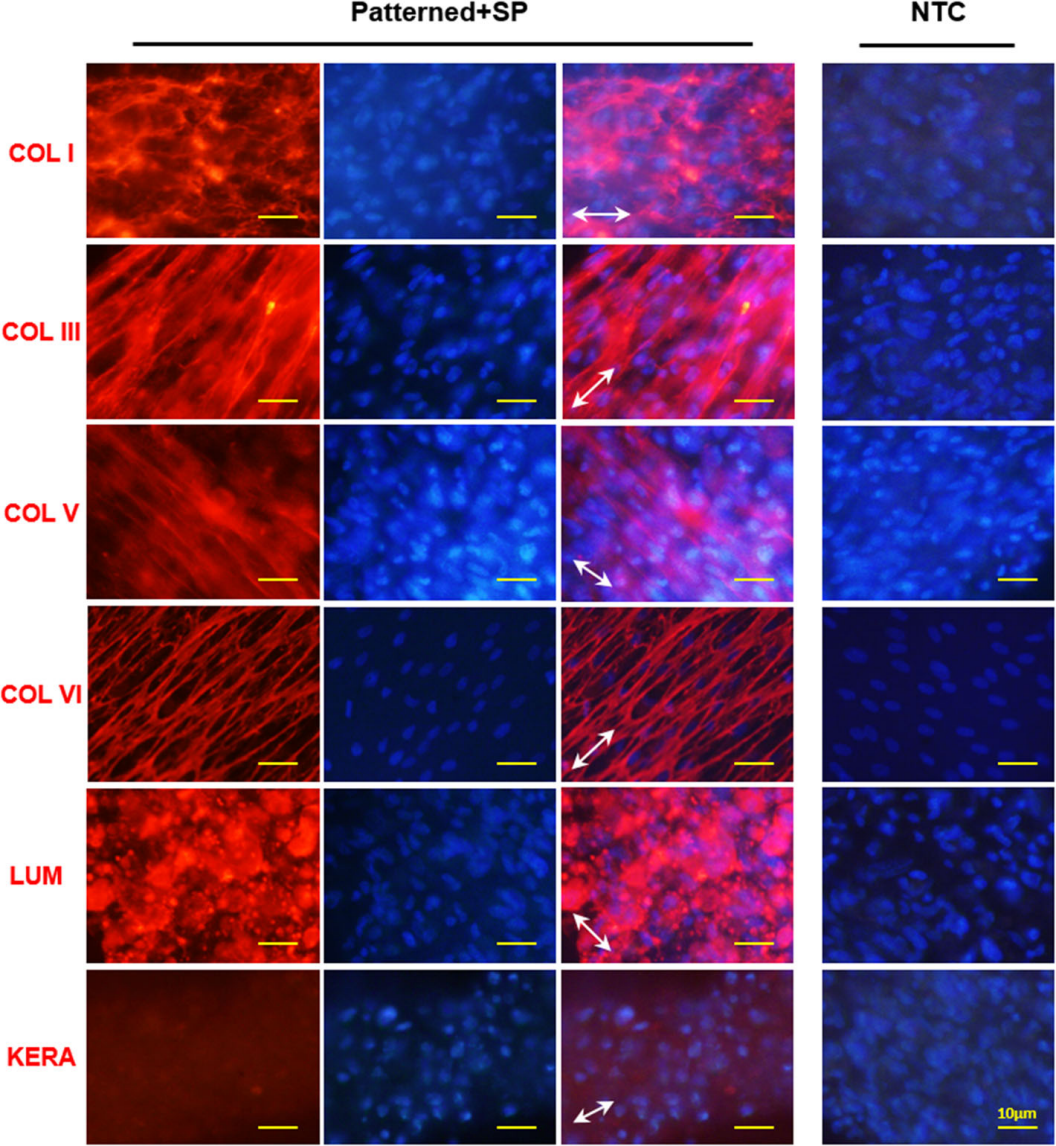
Stromal ECM components deposited on patterned silk membranes with substance P (*SP*). PDLSCs were seeded on patterned silk membranes and cultured in SP-supplemented keratocyte differentiation medium for 14 days. The expression of collagens and proteoglycans was evaluated by immunofluorescence staining. The *right panels* of the patterned + SP images show the merged picture of the *left panels* (ECM component staining) and the *middle panels* (DAPI staining). *White arrows* indicate the direction of the grooves on patterned silk membranes. The far *right panels* show the negative control (*NTC*) without primary antibody

After culturing PDLSCs on patterned silk membranes with SP-supplemented keratocyte differentiation medium for 14 days, the protein expression of typical ECM components of the corneal stroma was evaluated by immunofluorescence (Fig. 5). The expression of different collagens (COL I, COL III, COL V, and COL VI) and proteoglycans (LUM and KERA) was observed when compared to negative controls. In addition, the direction of these deposited proteins was along with the grooves of the silk membranes.

### Bioengineered human corneal stroma tissue constructs in vitro

**Fig. 6:**
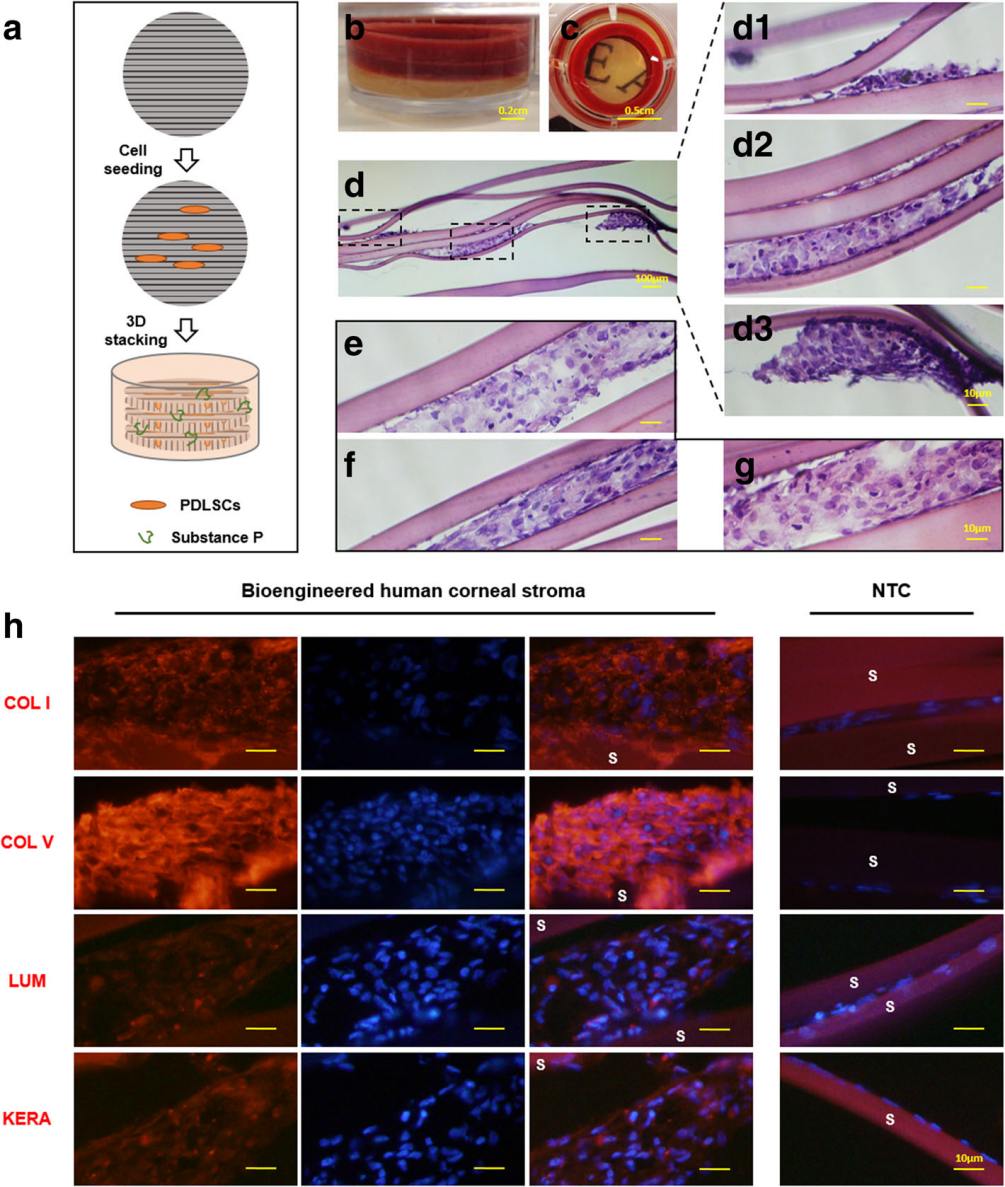
Bioengineered multi-lamellar human corneal stroma tissue constructs in vitro. **a** Schematic pictures of the assembly process for three-dimensional corneal stroma tissue constructs. Six patterned silk membranes with seeded PDLSCs (*orange cells*) were stacked one by one in a 24-well plate, with two plastic rings on the top of the construct (**b**) to keep the membranes close together. The constructs were cultured in keratocyte differentiation medium with 1 μM substance P (*green lines* in **a**) for 18 days. **c** Macroscopic picture shows that the constructs were clear enough to allow visualization of the letters below after 18 days of culture. **d** H&E staining showed the cells growing between every two layers (d1–d3). **e**–**g** Representative pictures show the fine integration of cells and silk membranes. **h** The expression of collagens and proteoglycans in bioengineered human corneal stroma was evaluated by immunofluorescence staining on frozen sections. The *right panels* of the bioengineered human corneal stroma images show the merged picture of the *left panels* (ECM component staining) and the *middle panels* (DAPI staining). The far *right panels* show the negative control (*NTC*) without primary antibody. *S* silk membrane

Bioengineered multi-lamellar human corneal stroma tissues were constructed in vitro by orthogonally stacking patterned silk membranes seeded with PDLSCs cultured in SP-supplemented KDM (Fig. 6a and b). The constructs were transparent after 18 days of culture (Fig. 6c). H&E staining was used to evaluate the structure of the assembly. Cells were found to grow between every two layers of silk membranes (Fig. 6d). Tissues were also formed between every two silk membranes (Fig. 6d1–d3), and integrated with the layers (Fig. 6e–g), although the structural integrity of the constructs was slightly destroyed by the freezing and sectioning procedure. The expression of corneal stroma ECM components (COL I, COL V, LUM, and KERA) was evaluated by immunofluorescence (Fig. 6h). High expression of COL I and COL V, the main collagen types in normal corneal stroma, was found in the in-vitro bioengineered stroma tissue. Important proteoglycans of normal corneal stroma (LUM and KERA) were also expressed in the constructs.

## Discussion

This study constructed a bioengineered multi-lamellar corneal stroma-like tissue in vitro by using PDLSCs as seeded cells, patterned silk membrane as scaffold, and substance P (SP) as a supplemental factor. Human PDLSCs were isolated and characterized, and could be efficiently differentiated into keratocytes with keratocyte differentiation medium. SP had no effect on the keratocyte differentiation, but promoted the expression of corneal stroma-related collagens (COL I, COL III, COL V, and COL VI). Patterned silk membranes guided cell alignment and supported corneal stroma ECM deposition (COL I, COL III, COL V, COL VI, LUM, and KERA) when seeded PDLSCs were cultured in keratocyte differentiation medium supplemented with SP. Based on this, multi-lamellar corneal stroma-like tissue was successfully constructed in vitro which could be beneficial for corneal tissue engineering and future corneal regeneration studies.

PDLSCs could be a potential cell source for keratocyte differentiation and corneal tissue engineering. Based on our results, PDLSCs can be efficiently differentiated into keratocytes with keratocyte differentiation medium. In addition, PDLSCs have several inherent advantages when compared to stem cells from other non-corneal tissues. Firstly, PDLSCs, as well as DPSCs, have the same neural crest origin as keratocytes in development, making them similar in proteoglycan secretion, and potentially beneficial for cell type transition between each other [9]. DPSCs have been reported capable of differentiating into keratocytes and to generate corneal stromal-like constructs [11]. However, to our knowledge, no similar evaluation has been previously carried out for PDLSCs. Secondly, PDLSCs are derived from periodontal ligaments. Tissues of ligaments/tendons have a high similarity to corneal stroma, both regarding physiological and pathological conditions. Normally, they both consist of aligned dense collagens, with COL I as the main component [26, 27]. Genome-wide gene expression pattern comparison has shown notable similarities in gene expression between tendons and corneas, especially in ECM collagens (collagen type I, III, V, and VI) and proteoglycan (lumican, decorin, and biglycan) [28]. In addition, both ligaments/tendons and corneas are subjected to physiological mechanical stress. When injured or degenerated, ligaments/tendons and corneal stroma often heal with scar formation, losing their well-organized collagen structures, with more cell accumulation and increased production of collagen type III relative to type I [29–31]. Thirdly, PDLSCs share the advantages of other dental stem cells, such as accessibility, high proliferation, and immunomodulatory properties [9, 32], which is promising for future clinical applications.

SP plays important roles in the cornea. Increased expression of *TAC1* (the gene coding for SP) and its receptor *TACR1* was found during the differentiation of PDLSCs towards keratocytes, but not in primary keratocytes (Fig. 2f and g). A promotion effect of SP on collagen expression instead of keratocyte markers was subsequently confirmed in our study. Interestingly, global genome-wide gene expression analysis by Wu et al. showed higher expression of ECM-related genes (including collagen types I, III, and VI) in the immature (postnatal day 10) mouse cornea as compared with adult mouse cornea [28], which indicates the demand for high collagen expression during corneal development. In our current model, upregulated SP and its receptor during the differentiation process (similar to the developmental process in vivo) contributed to the increased collagen expression. Furthermore, primary keratocytes in culture, like the mature adult cornea in vivo, do not need a high expression of collagens, which is consistent with the observed low expression of SP and its receptor. The promotion effect of SP on corneal stroma-related collagen expression is meaningful for in-vitro construction of three-dimensional corneal stroma tissue, with higher success rates and less time required. A previous report from our group also showed that SP enhanced collagen remodeling and expression of collagen type III in primary tendon cells, or tenocytes [33]. However, the effect of SP on collagen expression seems different in other cell types. It has been reported that SP inhibits collagen synthesis of rat myocardial fibroblasts [34] and human lung fibroblasts [35]. More work needs to be done in the future to elucidate the differences observed in different cell types.

Silk has advantages in mechanical strength, biocompatibility, and controllable biodegradability. Therefore, it has been widely used in multiple tissue engineering applications, including skin, bone, cartilage, tendon, cornea, and so forth [36–38]. Based on the well-organized collagen structures of normal cornea, aligned silk scaffolds have been designed and found to be efficient in supporting keratocyte proliferation, arrangement, and ECM deposition [39–42]. Nevertheless, most of these studies were only evaluated in a traditional two-dimensional model with merely one single silk membrane or silk film [10]. Since corneal stroma is a three-dimensional orthogonally aligned structure, it is important to construct bioengineered corneal stroma tissue with multi-layered aligned silk membranes to closely mimic the in-vivo corneal microenvironment. In 2009, Lawrence and collaborators reported a multi-layered film construct that was assembled with seven layers of porous/flat silk films with keratocytes seeded on each layer [39]. One year later, they improved this concept of three-dimensional constructs of corneal stroma with stacked arginine-glycine-aspartate (RGD)-coupled porous/patterned silk films [40]. The same group reported early this year that they had prepared three-dimensional functional corneal stromal tissue by orthogonally stacking aligned silk films seeded with human corneal stromal stem cells (hCSSCs) cultured for 9 weeks [43]. Our present study fabricated bioengineered human corneal stroma tissue in a similar way by stacking orthogonally aligned patterned silk membranes. With the advantages of PDLSCs, and the promotion effect of SP on the expression of collagens, our construct supports cell growth and new tissue formation between every two silk membranes over a relatively short culture period (18 days). High expression of the main collagen types found in normal human corneal stroma (COL I and especially COL V) was found in this bioengineered human corneal stroma tissue. The critical proteoglycans, LUM and KERA, were also expressed in the constructs. This new three-dimensional bioengineered human corneal stroma tissue model improves current corneal tissue engineering and shows a potential for future clinical applications. Meanwhile, by closely mimicking the microenvironment of in-vivo corneal stroma, this model is useful for evaluating cell behavior and function in vitro.

## Conclusions

In summary, the current study constructed a multi-lamellar corneal stroma-like tissue in vitro by using PDLSCs seeded on patterned silk membranes with SP supplementation. SP promoted corneal stroma-related collagen expression (COL I, COL III, COL V, and COL VI), and did not interfere with the induction effect of keratocyte differentiation medium (KDM). The patterned silk membrane directed cell alignment along the grooves and supported corneal stroma ECM deposition. Multi-lamellar corneal stroma-like tissue was assembled by orthogonally stacking six PDLSC-seeded patterned silk membranes cultured in SP-supplemented KDM. This in-vitro bioengineered multi-lamellar human corneal stroma tissue allowed cell growth between every two layers and supported the expression of the main type of collagens (COL I and COL V) and proteoglycans (LUM and KERA) of normal human corneal stroma, which indicates that this construct can be used as an in-vitro study model, as well as potentially being developed for clinical applications such as corneal transplantation in the future.

## Additional file

Additional file 1: Figure S1. Substance P promotes collagen expression during induced keratocyte differentiation. (A) Immunofluorescence staining was carried out to compare the collagen expression between the control (Ctrl) and SP-treated groups (SP). The right panels are the merged picture of the left panels (collagen staining) and the middle panels (DAPI staining). (B) The integrated density of fluorescence in the different groups was quantified using ImageJ analysis software. The integrated density levels were higher in SP-treated groups. However, no significant difference was found (*p* ≥ 0.05). (PDF 138 kb)

## Abbreviations

A2-P: l-Ascorbic acid 2-phosphate
ACTB: β-Actin
ALDH1A1: Aldehyde dehydrogenase 1A1
ALDH3A1: Aldehyde dehydrogenase 3A1
ASC: Adipose-derived stem cell
BGN: Biglycan
BM-MSC: Bone marrow-derived mesenchymal stem cell
COL I: Collagen type I
COL III: Collagen type III
COL V: Collagen type V
COL VI: Collagen type VI
COL XIV: Collagen type XIV
CSSC: Corneal stromal stem cell
DMEM: Dulbecco’s modified Eagle’s medium
DPSC: Dental pulp stem cell
ECM: Extracellular matrix
EPHA4: EPH receptor A4
FBS: Fetal bovine serum
GAPDH: Glyceraldehyde 3-phosphate dehydrogenase
H&E: Hematoxylin and eosin
KDM: Keratocyte differentiation medium
KERA: Keratocan
LUM: Lumican
MEM-α: minimum essential medium-α
MKX: Mohawk
NFATC4: Nuclear factor of activated T-cells 4
PDLSC: Periodontal ligament stem cell
qPCR: Quantitative polymerase chain reaction
RGD: Arginine-glycine-aspartate
SCX: Scleraxis
SEM: Scanning electron microscope
SP: Substance P
TAC1: Tachykinin precursor 1
TACR1: Tachykinin receptor 1
UMSC: Umbilical cord-derived mesenchymal stem cell
