# Supplementary material for: Substance P and patterned silk biomaterial stimulate periodontal ligament stem cells to form corneal stroma in a bioengineered three-dimensional model
Source: Stem Cell Res Ther. 2017 Nov 13;8:260. doi: 10.1186/s13287-017-0715-y (PMC5683543; doi:10.1186/s13287-017-0715-y)
Supplement: Additional file 1: Figure S1. — Substance P promotes collagen expression during induced keratocyte differentiation. (A) Immunofluorescence staining was carried out to compare the collagen expression between the control (Ctrl) and SP-treated groups (SP). The right panels are the merged picture of the left panels (collagen staining) and the middle panels (DAPI staining). (B) The integrated density of fluorescence in the different groups was quantified using ImageJ analysis software. The integrated density levels were higher in SP-treated groups. However, no significant difference was found (p ≥ 0.05). (PDF 138 kb) [file 13287_2017_715_MOESM1_ESM.pdf]

Substance P and Patterned Silk Biomaterial Stimulate Periodontal Ligament Stem Cells to Form Corneal Stroma in a Bioengineered 3D Model

Jialin Chen[a,1]; Wei Zhang[a,1]; Peyman Kelk[a]; Ludvig J. Backman[a, b]; Patrik Danielson[a,c,*]

[a] Department of Integrative Medical Biology, Anatomy, Umeå University, Umeå, Sweden

[b] Department of Community Medicine and Rehabilitation, Physiotherapy, Umeå University, Umeå, Sweden

[c] Department of Clinical Sciences, Ophthalmology, Umeå University, Umeå, Sweden

[1] These authors contributed equally.

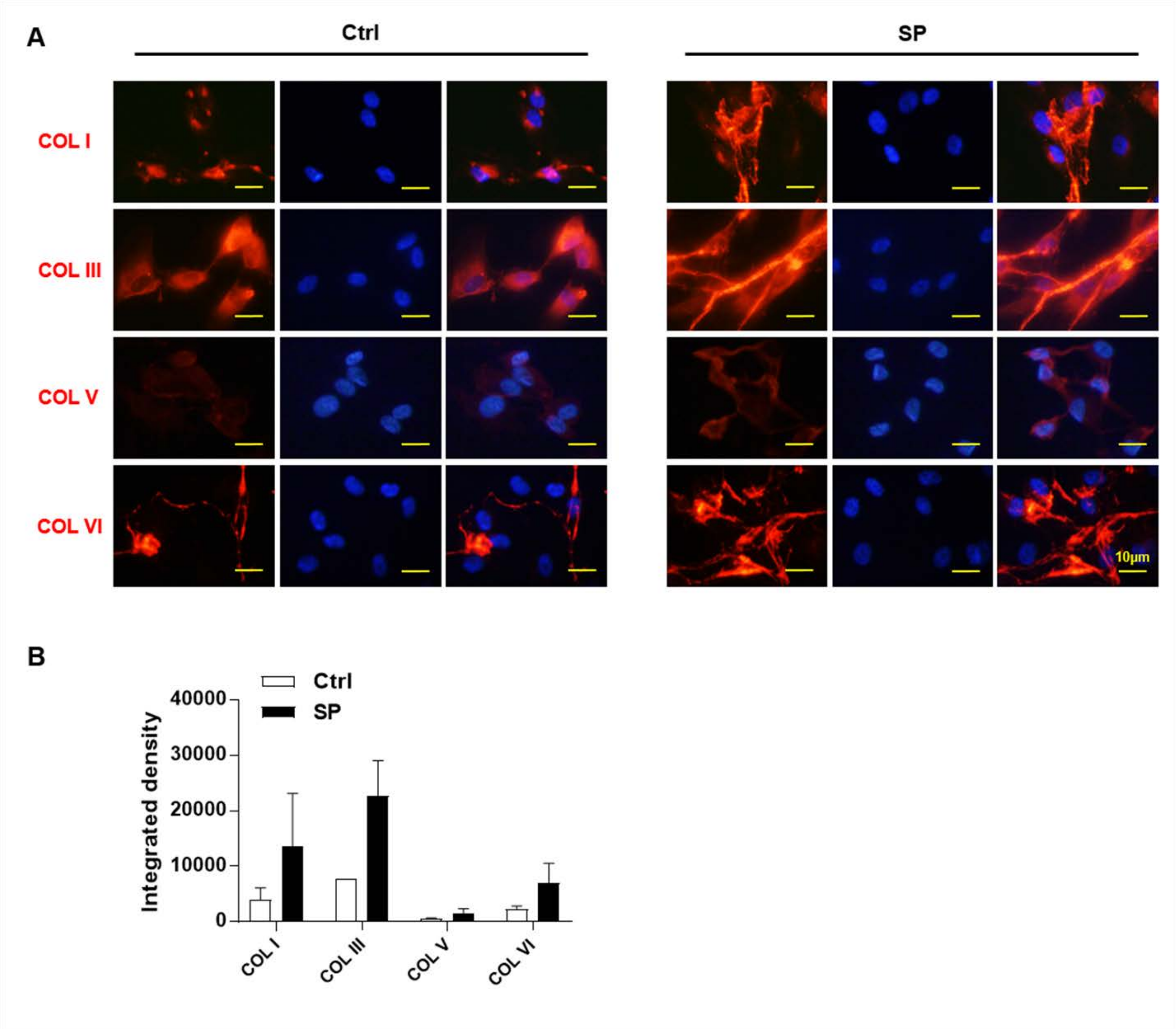

Supplementary Figure 1. Substance P promotes collagen expression during induced keratocyte differentiation. (A) Immunofluorescence staining was carried out to compare the collagen expression between control (Ctrl) and SP-treated group (SP). The right column is the merged picture of left column (collagen staining) and middle column (DAPI staining). (B) The integrated density of fluorescence in different groups was quantified using ImageJ analysis software. The integrated density levels were higher in SP-treated groups. However, no significant difference was found ($p \geq 0.05$).
